# Acrylamide exposure aggravates the development of ulcerative colitis in mice through activation of NF-κB, inflammatory cytokines, iNOS, and oxidative stress

**DOI:** 10.22038/ijbms.2021.52233.11816

**Published:** 2021-03

**Authors:** Keyvan Amirshahrokhi

**Affiliations:** Department of Pharmacology, School of Pharmacy, Ardabil University of Medical Sciences, Ardabil, Iran

**Keywords:** Acrylamide, Apoptosis, Cytokines, Inflammation, Oxidative stress, Ulcerative colitis

## Abstract

**Objective(s):**

Acrylamide is a toxic compound that forms during food processing at high temperatures. Acrylamide has been shown to induce toxicity in various organs in the body. This study aimed to investigate the effect of acrylamide exposure on the susceptibility of the colon to ulcerative colitis in a mouse model.

**Materials and Methods:**

Mice were pretreated with acrylamide (oral, 20 and 30 mg/kg/day) for 21 consecutive days, and colitis was induced by intrarectal administration of acetic acid.

**Results:**

The results revealed that acrylamide-pretreatment significantly increased disease activity index (DAI), macroscopic damage, histological changes of the colonic mucosa and oxidative stress markers carbonyl protein, malondialdehyde (MDA), and nitric oxide (NO), whereas it decreased the levels of anti-oxidants glutathione (GSH), superoxide dismutase (SOD) and catalase. Moreover, induction of colitis in acrylamide-pretreated mice caused a higher increase in colonic levels of myeloperoxidase (MPO), matrix metalloproteinase (MMP)-9, monocyte chemoattractant protein (MCP)-1, cytochrome-c, caspase-3, proinflammatory cytokine tumor necrosis factor (TNF)-α, interleukin (IL)-6, IL-1β, and interferon (IFN)-γ, whereas it reduced the level of IL-10. The mRNA expression of nuclear factor kappa B (NF-κB) and inducible nitric oxide synthase (iNOS) were further increased in colon tissue of mice exposed to acrylamide.

**Conclusion:**

These findings suggest that acrylamide can accelerate the development of acetic acid-induced colitis. In conclusion, chronic acrylamide exposure may aggravate the severity of ulcerative colitis and increase colonic mucosal damage through oxidative stress and inflammatory responses.

## Introduction

Acrylamide (or acrylic amide) is a chemical compound that is widely used in various industrial applications. It is used in the production of paper, plastics and textiles, and treatment of waste and drinking water. Acrylamide can be found in small amounts in some cosmetics, personal care products, and tobacco smoke however the main source of acrylamide to humans is some foods. Acrylamide is formed in the reaction between the amino residue of the amino acid asparagine and the carbonyl from a reducing sugar during high-temperature food processing. The main food sources of acrylamide are starchy foods such as potato chips, French fries, bread, cookies, and coffee that has been heated to high temperatures (above 120 °C) (1, 2). Acrylamide is an extremely toxic and potentially carcinogenic substance that can be absorbed through the skin and gastrointestinal and respiratory tracts. After absorption, acrylamide is rapidly distributed to various tissues and metabolized to glycidamide, which is a highly reactive epoxide. Acrylamide has been reported to cause toxicity in the CNS, liver, kidney, reproductive system, and gastrointestinal tract (1, 3, 4). It has been shown that acrylamide causes damage to cells in different ways, including inflammatory responses, oxidative stress, apoptosis, and DNA damage. It has been reported that the inflammatory cytokines TNF-α, IL-1β, and IL-6 were increased during acrylamide-induced toxicity. It has also been shown that acrylamide activates nuclear factor-κB signaling pathway and stimulates inducible form of NOS (5, 6). After oral consumption, acrylamide is readily absorbed by the gastrointestinal tract; therefore the intestinal tissue is exposed to a high concentration of acrylamide. Indeed intestinal tissue is one of the main targets of acrylamide-induced toxicity. Acrylamide has been reported to cause gastric and intestinal mucosal injury by increasing the inflammatory responses, oxidative stress and apoptosis (7-9). 

Ulcerative colitis is an intestinal inflammatory disease with chronic inflammation and ulceration of the intestine. The most common signs of ulcerative colitis are abdominal pain, bloody diarrhea and weight loss. It has been well known that patients with colitis have a high risk for the development of colorectal cancer. Several factors, such as dysregulated immune response, environmental factors, genetics, intestinal microbiota and intestinal mucosal barrier damage are involved in the pathogenesis of colitis. Excessive production of reactive oxygen species, nitric oxide and proinflammatory cytokines, including IL-1β, TNF-α, IL-6 and MCP-1 can produce oxidative and inflammatory damages to the intestinal mucosa. These biological processes are controlled by NF-κB, which is a key transcription factor involved in the development of ulcerative colitis (10-12). 

Numerous environmental agents are able to disturb the host immune response and the integrity of the intestinal epithelial barrier. Diet, food additives and cigarette smoking influence the development of colitis. Toxic agents in food such as bacterial peptides have long been associated with the onset of inflammatory bowel diseases (13, 14). Acrylamide is one of the most important food toxicants. It has been shown that acrylamide exposure can lead to gastric and intestinal mucosal injury through induction of oxidative and inflammatory responses; however, the relationship between acrylamide and ulcerative colitis has remained unclear. The aim of our study was to elucidate the possible association between exposure to moderate and high doses of acrylamide and susceptibility to colitis in an experimental model.

## Materials and Methods

***Animals***

The experiments were carried out in male mice (NMRI) weighing 21–25 g. Mice were housed in a standard animal house with free access to water and laboratory food. Before the induction of colitis, animals were fasted overnight with access to water. All experiments were done according to the NIH Guide for the Care and Use of Laboratory Animals and by the approval of our institutional Ethics Committee (IR.ARUMS.REC.1398.605).

***Colitis induction***

Before the induction of colitis, animals were slightly anesthetized by ether and a flexible catheter was inserted into the rectum until the tip was 4 cm proximal to the anus. Experimental colitis was induced by a single intrarectal administration of acetic acid (5%, 150 µl) (10-11).

***Design of the Experiments***

The mice were allocated into four experimental groups (n = 10); (1) group of normal mice received saline solution. (2) Control colitis group mice received intrarectal acetic acid. (3) AA (20) group mice were pretreated with acrylamide (20 mg/kg/day, oral) and received intrarectal acetic acid. (4) AA (30) group mice were pretreated with acrylamide (30 mg/kg/day, oral) and received intrarectal acetic acid. The doses of acrylamide were selected based on a prior study (7) and our pilot experiments. Acrylamide was dissolved in normal saline and gavaged once daily for 21 consecutive days. Colitis was induced on day 22 by single intrarectal instillation of acetic acid. At 48 hr after colitis induction, mice were anesthetized with xylazine and ketamine and the colon was dissected. After macroscopic examination, colon tissues were frozen in liquid nitrogen and retained at −70 °C. 

***Assessment of disease activity index (DAI)***

After induction of colitis, body weight, condition of stool, and rectal bleeding were recorded in mice. DAI was calculated as the total mean score for body weight loss, stool consistency and colonic gross bleeding (Table 1).

***Macroscopic examination***

The entire length of each colon was measured. Then, the colon was longitudinally opened and washed by cold saline solution. The macroscopic injury of the colonic mucosa was evaluated and scored based on the following criteria: 0 = no damage; 1 = mucosal erythema only; 2 = mild edema, slight bleeding; 3 = moderate edema, bleeding ulcers; 4 = severe edema, ulceration and tissue necrosis (11). 

***Histological analysis***

A portion of each resected colon tissue was fixed in formalin (10%). The paraffinized-tissue blocks were sectioned and stained with Hematoxylin and Eosin (H&E). The histopathological changes of the colon were assessed and scored by a pathologist according to the following criteria: submucosal edema, damage or necrosis, vasculitis and inflammatory cell infiltration. Each criteria was scored on a scale from 0 to 3 according to the severity of changes (0 = none; 1 = slight; 2 = moderate; 3 = severe) (10). 

***Homogenization of colon tissues***

Frozen colon tissues were cut into small parts and homogenized with a homogenizer (Heidolph, Germany) in Trisma buffer (pH 7.4) containing complete protease inhibitor (Roche). The homogenized samples were centrifuged at 20,000×g for 20 min at 4 °C and their supernatants were separated from the pellets and stored at -70 °C for biochemical analysis. 

***Measurement of colonic MDA, carbonyl proteins, GSH, catalase. and MPO***

The colonic tissue supernatants were used to evaluate oxidative stress and inflammatory markers. MDA concentration was assessed by the method of thiobarbituric acid (TBA) reaction. This method is based on the reaction of TBA with MDA to produce a color complex which can be measured spectrophotometrically at 532 nm. The carbonyl protein content was measured by 2,4-dinitrophenylhydrazine (DNPH) reaction. This method is based on the spectrophotometric detection of the reaction of DNPH with protein carbonyls to produce protein hydrazones with absorbance at 370 nm. The level of reduced glutathione (GSH) was assessed using Ellman’s reagent (5,5′-dithiobis-2-nitrobenzoic acid; DTNB). The test is based on the reaction of DTNB with GSH to produce a yellow compound with a maximum absorbance at 412 nm. Catalase activity was assessed by decomposition of hydrogen peroxide (H_2_O_2_) to water and oxygen. The rate of change in absorbance was recorded at 240 nm. One unit of catalase activity was defined as the quantity of the enzyme that decomposes 1 mM of H_2_O_2_ per minute. MPO activity was evaluated by the process of H_2_O_2_–dependent oxidation of tetramethylbenzidine (TMB). In the presence of MPO, oxidation of TMB produces a blue colored product that can be measured at 370 nm (15). The results were expressed as per mg protein content of the supernatants. 

***Measurement of colonic SOD, NO, MMP-9, cytochrome-c, and caspase-3***

The level of SOD in the colonic tissue supernatants was assessed by an SOD assay kit (Cayman Chemical). The concentrations of nitrite and nitrate as markers of nitric oxide production were assessed by an NO Assay Kit (Cayman Chemical). The level of MMP-9 was evaluated by an ELISA kit (Mouse Total MMP-9 Kit, R&D Systems). Mouse Cytochrome-C and CASPASE-3 ELISA kits (ZellBio Gmbh, Germany) were used to assay cytochrome-c and caspase-3 according to their instructions for use.

***Real-time PCR analysis of iNOS and NF-*** ***κB***

Tissue RNA was extracted by Trizol reagent (Roche). Total RNA was converted to cDNA using a First-Strand cDNA Synthesis Kit (Thermo Scientific, USA). Synthesized first-strand cDNA was used for SYBR Green-based real time PCR analysis by a LightCycler (Roche). The PCR cycling started with an initial incubation (95 °C, 5 min) followed by 40 amplification cycles (94 °C for 10 sec, 59 °C for 15 sec) and a final extension (72 °C, 10 min). GAPDH as a housekeeping gene was used to normalize the amount of expression levels. The sequences of primers were as follows: NF-κB forward, ACCTTTGCTGGAAACACACC and reverse, ATGGCCTCGGAAGTTTCTTT; GAPDH forward,CTGCCACCC

AGAAGACTGTG and reverse, GGTCCTCAGTGTAGCCCAAG; iNOS forward, GCCTTGGCTCCAGCATGTACCCTCAG and reverse, CCTGCCCACTGAGTTCGTCCCCTTC.

***Evaluation of colonic cytokines***

The levels of inflammatory cytokines in the supernatants of colon tissue were assessed by mouse ELISA kits for cytokines IL-1β, TNF-α, IL-6, IFN-γ, and IL-10 (eBioscience) according to the manufacturer’s instructions. The chemokine MCP-1 was assessed using a Mouse MCP-1 ELISA kit (ZellBio Gmbh, Germany). The concentrations were expressed as ng or pg/mg protein.

***Statistical analysis***

Data were expressed as mean±standard error (SEM). Significant differences among groups were calculated by ANOVA (one-way analysis of variance) followed by Tukey’s *post-hoc* test. Histopathological and macroscopic scores were evaluated by non-parametric Kruskal–Wallis test followed by Dunn’s *post-hoc* test, and *P*<0.05 was considered as statistically significant. 

## Results

***Effect of acrylamide pretreatment on the body weight changes***

**Figure 1:**
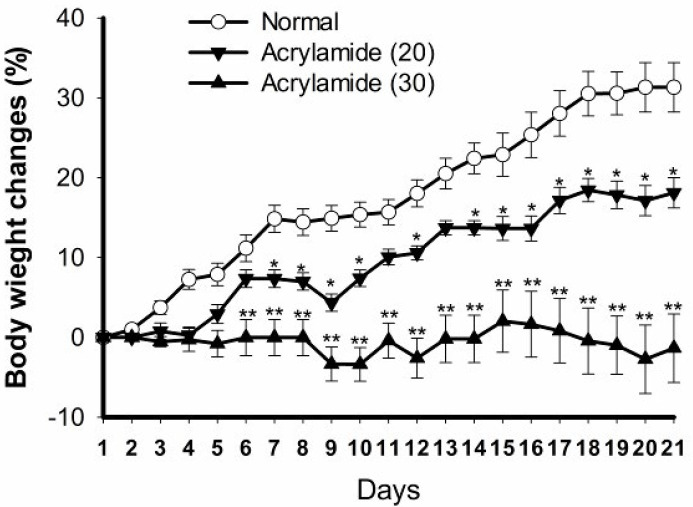
Effect of acrylamide-pretreatment on body weight changes (%). Compared with the normal saline-treated mice, oral administration of acrylamide (20 and 30 mg/kg/day, for 21 days) decreased the body weight of mice during pretreatment. Data are means±SEM, (n=10). ^*^P<0.05,^ **^P<0.01 vs normal group

The changes of body weight were measured daily during the course of acrylamide pretreatment. As shown in Figure 1, compared with normal saline-treated mice, oral administration of acrylamide (20 and 30 mg/kg/day) caused a decrease in body weight of mice during 21 days of pretreatment. 

***Effect of acrylamide on total DAI score, length of colon, and mortality rate in colitis***

**Figure 2:**
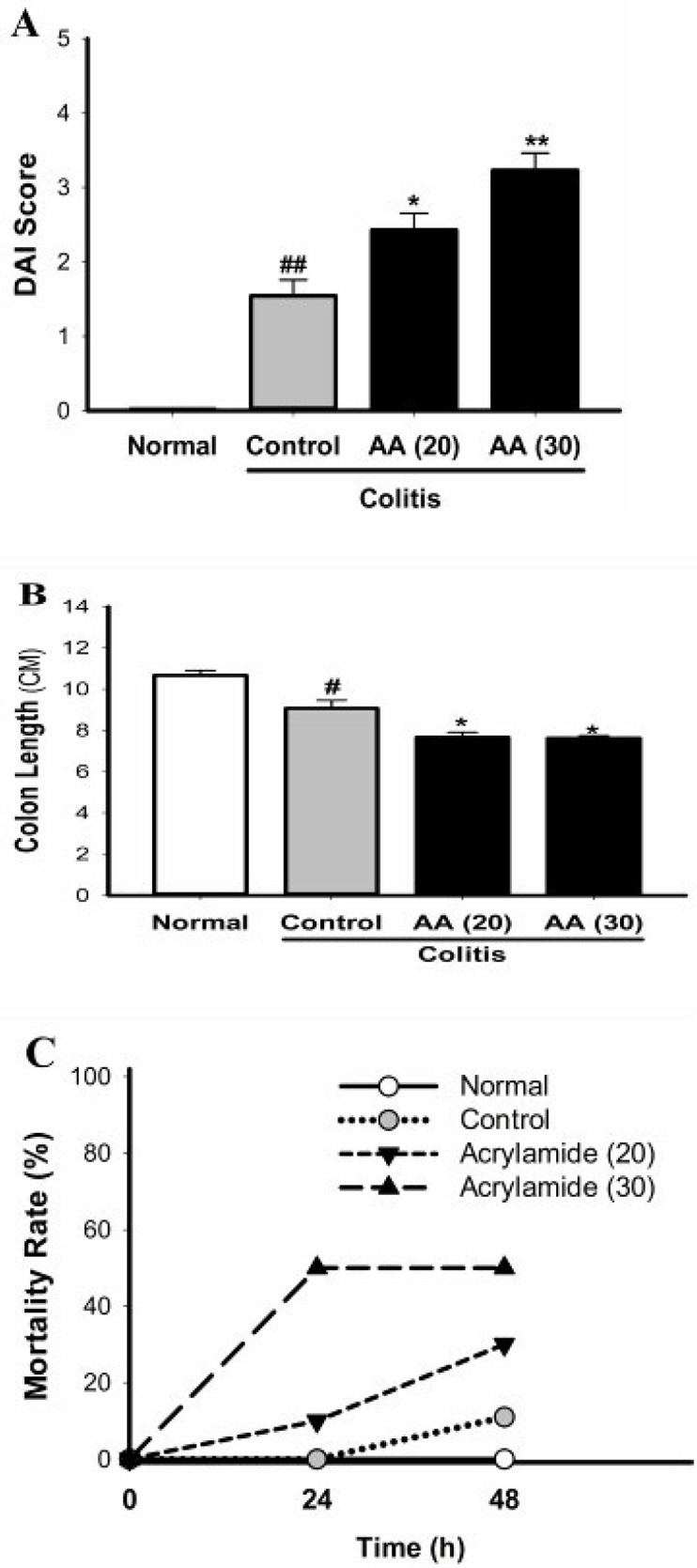
Effect of acrylamide on DAI score (A), length of colon (B), and mortality rate of mice (C), in acetic acid-induced colitis. The total DAI score, colon shortening and mortality rate were significantly increased in colitis mice pretreated with acrylamide. Data are means±SEM. (n=10). ^#^*P*<0.05, ^##^*P*<0.001 vs normal group; ^*^P<0.05, ^**^P<0.01 vs control group

After induction of colitis in mice the clinical signs of colitis were determined by assessing DAI (Figure 2A). Compared with the normal mice, the total DAI score of all colitis groups was considerably increased. However in both groups pretreated with acrylamide, DAI as an indicator of the severity of colitis was higher than that in control mice. Acrylamide at the dose of 30 mg/kg was more effective than that at the dose of 20 mg/kg. Shortening of colon length is considered an indicator of colonic inflammation and damage. Intrarectal use of acetic acid reduced the length of colon in the control group (Figure 2B). The colon length in mice pretreated with acrylamide was shorter than that in the control mice. As shown in Figure 2C, administration of acetic acid in the control group resulted in mortality rate of 10 % while induction of colitis in mice exposed to acrylamide (20 and 30 mg/kg/day) caused a higher rate of mortality (30% and 50%, respectively). 

***Effect of acrylamide on the macroscopic appearance in colitis***

**Figure 3:**
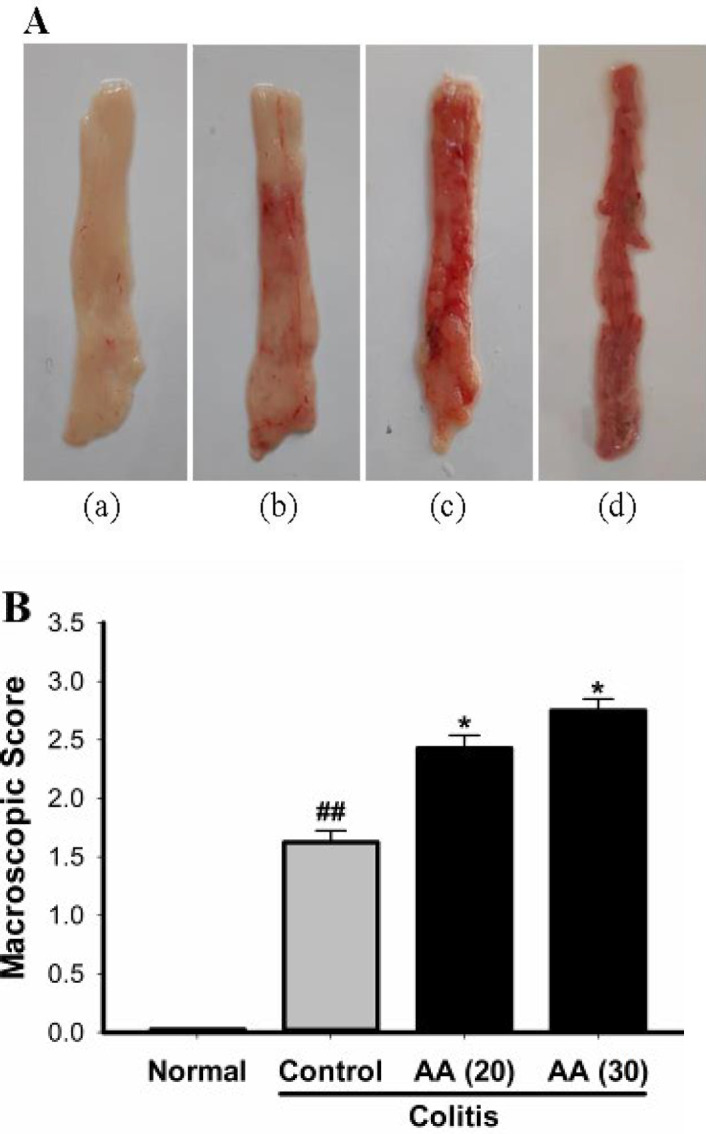
(A) Macroscopic features of colon mucosa in normal (a), colitis control (b), colitis acrylamide (20 mg/kg/day) (c), and colitis acrylamide (30 mg/kg/day) (d). The colonic mucosal damage was aggravated in colitis mice exposed to acrylamide. (B) Effect of acrylamide pretreatment on macroscopic scores of colonic mucosal damage. Results were analyzed by non-parametric Kruskal–Wallis test. Results are means±SEM. ^##^*P*<0.001 vs normal group; ^*^P<0.05 vs control group

The colonic mucosa in saline-treated mice was normal without any damage (Figure 3A). Administration of acetic acid into the colon of mice produced mucosal injury as revealed by edema, hyperemia, slight bleeding, and small mucosal erosions. These changes in the colonic mucosa confirmed the induction of ulcerative colitis and resulted in increase in mean macroscopic score as compared with normal mice. These morphological changes in the colonic mucosa and macroscopic scores in mice exposed to acrylamide were higher than those in control mice (Figures 3A and B). These data indicate that acrylamide was able to increase the susceptibility of colonic mucosa to acetic acid-induced colitis.

***Effect of acrylamide on pathological changes in colitis***

**Figure 4:**
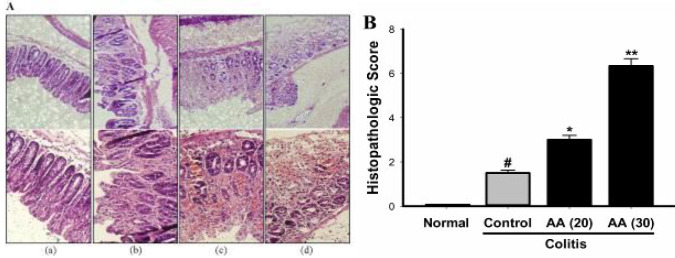
(A) Histological appearance of colon tissue in normal (a), colitis control (b), colitis acrylamide (20 mg/kg/day) (c), and colitis acrylamide (30 mg/kg/day) (d). The histological changes of the colon tissue in mice pretreated with acrylamide were significantly intensified. (B) Effect of acrylamide pretreatment on histopathological scores in colon tissue of colitis mice. Results were analyzed by non-parametric Kruskal–Wallis test. Results are means±SEM. ^#^*P*<0.05 vs normal group; ^*^P<0.05, ^**^P<0.01 vs control group

Histopathological analysis of colon tissue in saline treated mice exposed intact epithelium with no histological abnormalities (Figure 4A). In contrast, in control colitis mice intrarectal use of acetic acid produced an inflammatory reaction characterized by submucosal edema, leukocyte infiltration, epithelial damage, and loss of goblet cells. These histopathological alterations of colon mucosa in mice treated with acrylamide were significantly intensified. As shown in Figure 4B the histopathological score was also increased in both acrylamide groups as compared with the control colitis mice. Aggravating effect of acrylamide on the histological features of colon at dose of 30 mg/kg was significantly higher than that at dose of 20 mg/kg.

***Effect of acrylamide on*** ***MDA, protein carbonyl,*** ***SOD, GSH, and catalase levels***

**Figure 5:**
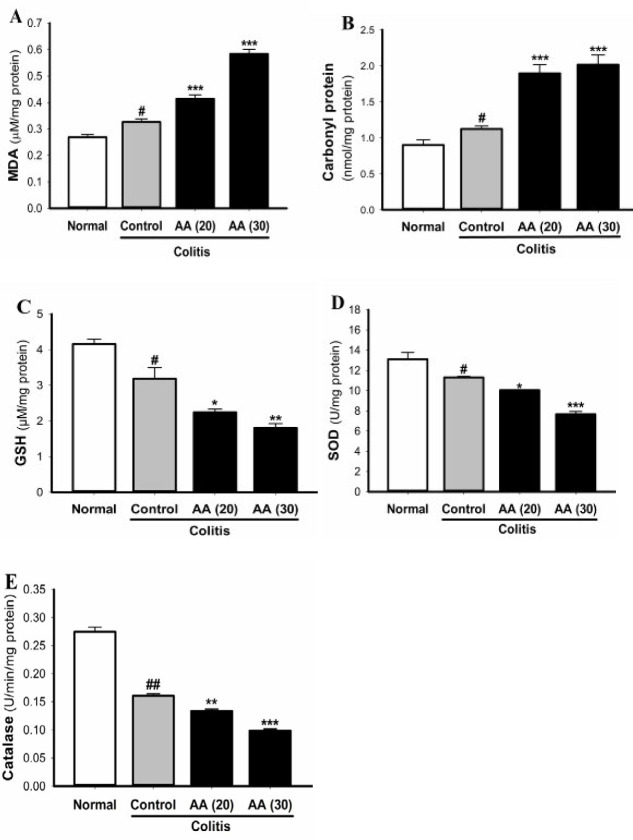
Effect of acrylamide on MDA (A), protein carbonyl (B), GSH (C), SOD (D), and catalase (E) levels in colonic tissue. Results are means±SEM, (n=5-7). ^#^*P*<0.05, ^##^*P*<0.001 vs normal group; ^*^P<0.05, ^**^P<0.01, ^***^P<0.001 vs control group

MDA: malondialdehyde; GSH: glutathione; SOD: superoxide dismutase

MDA and carbonyl protein levels of colon tissue were evaluated as biochemical index of lipid peroxidation and protein oxidation. As shown in Figures 5A and B, after the induction of colitis, MDA and protein carbonyl levels were increased. The elevation of MDA and carbonyl protein in colon tissue of mice exposed to acrylamide was significantly increased as compared with the control group. Acetic acid-induced oxidative stress in colon tissue leads to the depletion of SOD, GSH, and catalase as endogenous anti-oxidants (Figures 5C, D, and E). The reduction of SOD, GSH, and catalase was significantly intensified in both acrylamide groups (20 and 30 mg/kg/day) as compared with those in the control mice. The effect of acrylamide on the biomarkers MDA, SOD, GSH, and catalase was dose dependent. Therefore, chronic acrylamide exposure impairs the anti-oxidant capacity of the colon during colitis. 

***Effect of acrylamide on*** ***the level of NO and the expression of iNOS***

**Figure 6:**
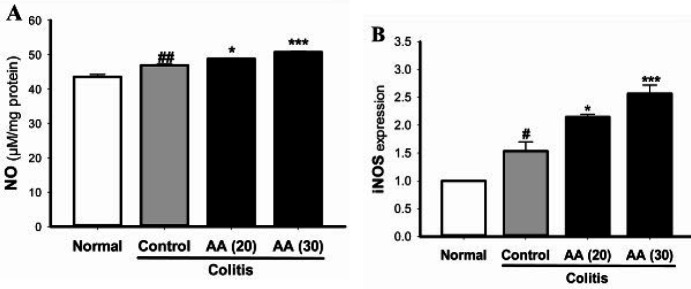
Effect of acrylamide on production of NO (A) in colonic tissue of colitis mice. Data are means±SEM, (n=5–6). Effect of acrylamide-pretreatment on iNOS mRNA expression (B) in colon tissue of colitis mice. GAPDH was used to normalize mRNA expression. Results are means±SEM, (n=4-5). ^#^*P*<0.05, ^##^*P*<0.001 vs normal group; ^*^P<0.05, ^***^P<0.001 vs control group

As shown in Figures 6A and B, acetic acid-induced ulcerative colitis increased iNOS expression and the production of NO in colon tissue. Compared with the control group, chronic pretreatment of mice with acrylamide caused a further increase in iNOS expression and subsequently NO production in colon tissue during colitis. 

***Effect of acrylamide on*** ***MPO, MMP-9, and MCP-1 levels***

**Figure 7:**
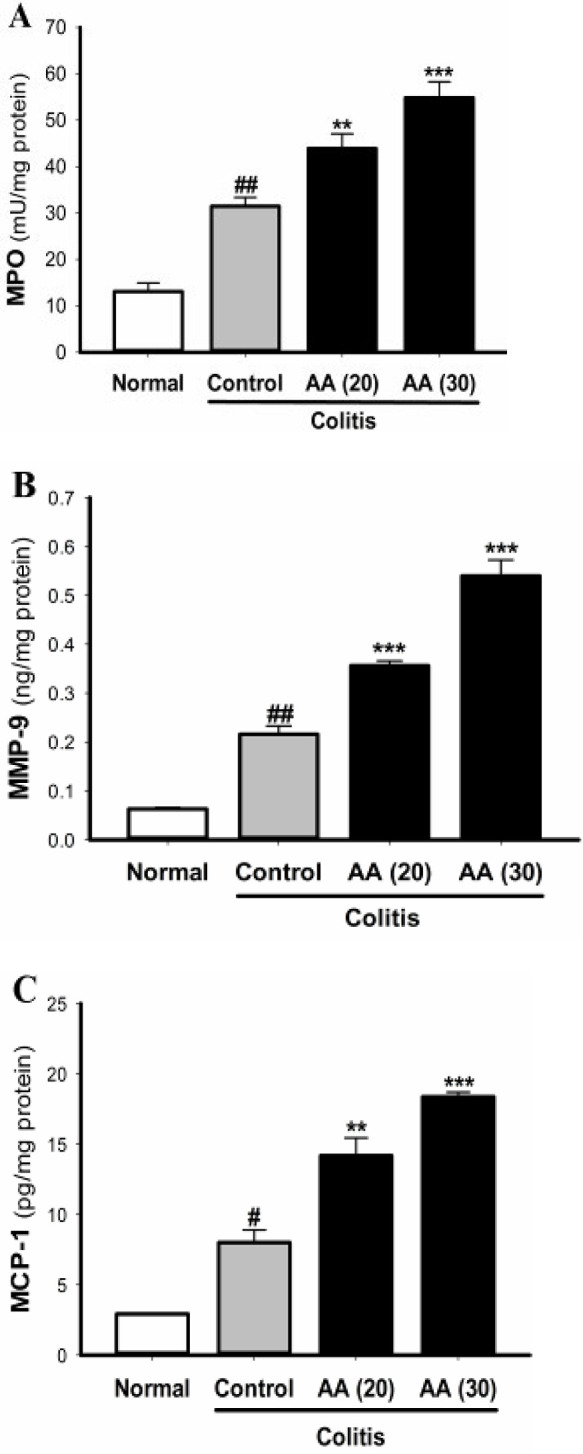
Effect of acrylamide on MPO (A), MMP-9 (B), and MCP-1 (C) levels in colon tissue of colitis mice. Data are means±SEM, (n=5-7). ^#^*P*<0.05, ^##^*P*<0.001 vs normal group; ^**^P<0.01, ^***^P<0.001 vs control group

MPO: myeloperoxidase; MMP: matrix metalloproteinases; MCP: monocyte chemoattractant protein

Experimental colitis increased the activity of MPO and the levels of MCP-1 and MMP-9 as markers of inflammatory damage in the colonic tissue (Figure 7). The elevation in the colonic contents of MPO, MMP-9, and MCP-1 was intensified in mice exposed to acrylamide as compared with the control colitis mice. The effect of acrylamide on the levels of MPO, MMP-9, and MCP-1 at the dose of 30 mg/kg was higher than that at dose of 20 mg/kg. This result indicates that chronic acrylamide exposure could stimulate colitis-induced immune cell infiltration and colonic epithelium destruction. Moreover, this result was consistent with the histopathological findings.

***Effect of acrylamide on*** ***the level of cytochrome-c and caspase-3***

**Figure 8:**
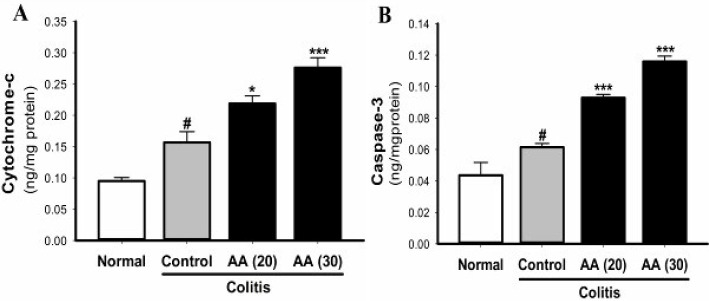
Effect of acrylamide on cytochrome-c (A) and caspase-3 (B) levels in colon tissue of colitis mice. Data are means±SEM, (n=5). ^#^*P*<0.05 vs normal group; ^*^P<0.05, ^***^P<0.001 vs control group

As shown in Figure 8, induction of colitis increased the levels of cytochrome-c and caspase-3 as makers of apoptosis in colon tissues of colitic mice when compared with the normal saline-treated mice. The elevation in the colonic level of cytochrome-c and caspase-3 was dose-dependently increased in mice pretreated with acrylamide (20 and 30 mg/kg) as compared with the control colitis mice.

***Effect of acrylamide on NF-*** ***κB*** ***expression and the inflammatory cytokines***

**Figure 9:**
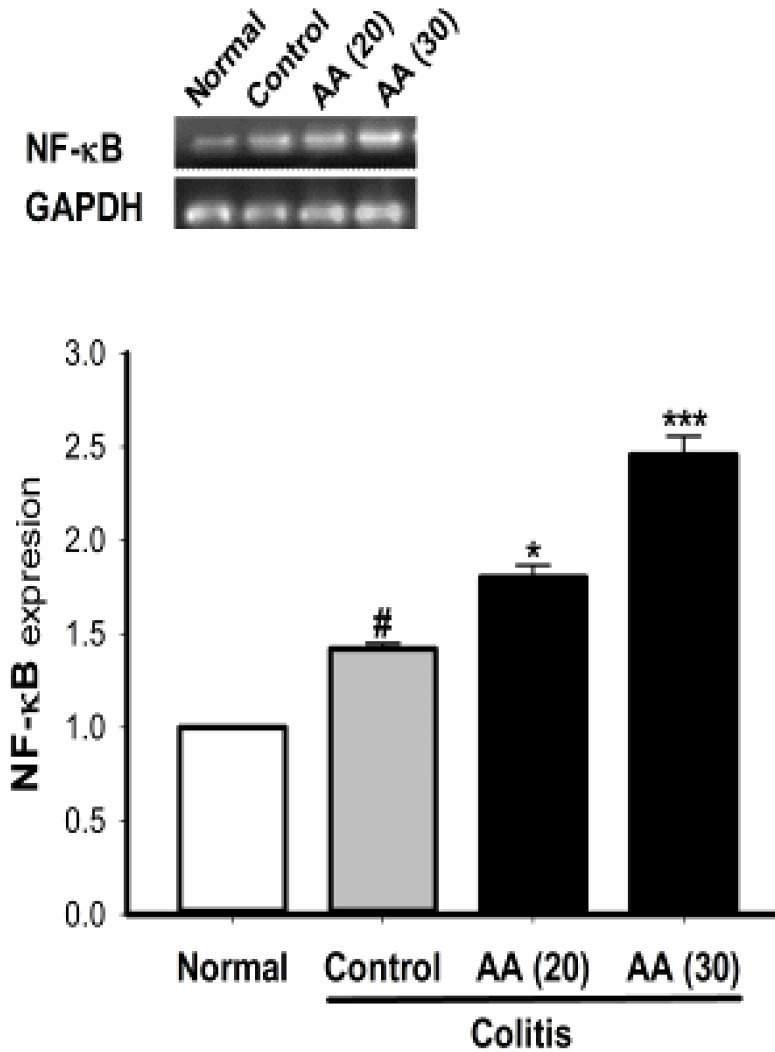
Effect of acrylamide on NF-κB mRNA expression in the colon of colitis mice. GAPDH was used to normalize mRNA expression. Results are means±SEM, (n=3). ^#^*P*<0.01 vs normal group; ^*^P=0.01, ***P<0.001 vs control group

**Figure 10:**
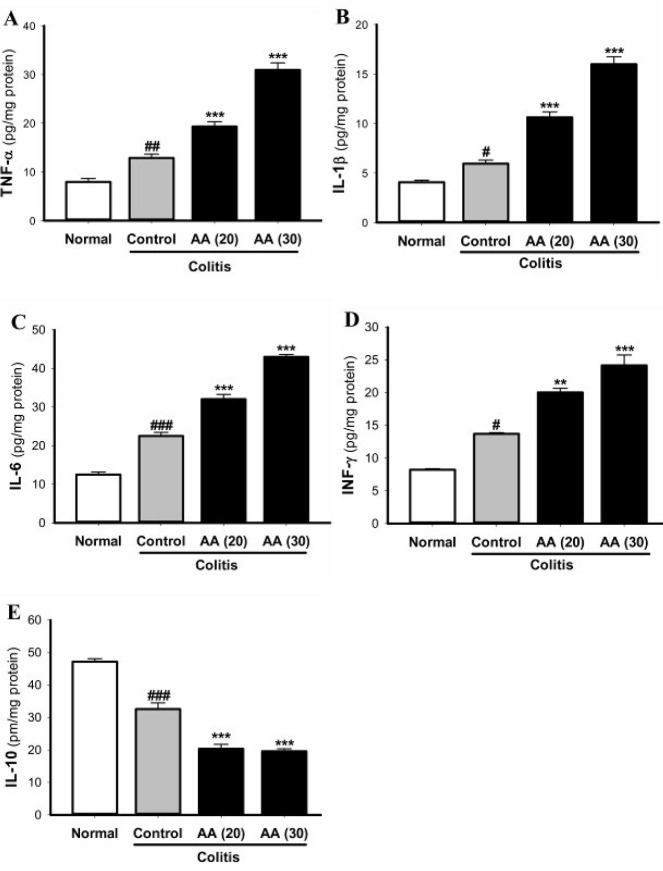
Effect of acrylamide on cytokines TNF-α (A), IL-1β (B), IL-6 (C), IFN-γ (D), and IL-10 (E) levels in colon tissue of colitis mice. Results are means±SEM, (n=5–7). ^#^*P*<0.05, ^##^*P*<0.01, ^###^*P*<0.001 vs normal group; ^*^P<0.05, ^**^P<0.01, ^***^P<0.001 vs control group

Evaluation of NF-κB gene expression in the experimental groups revealed that induction of colitis increased the expression of NF-κB in all colitis mice (Figure 9). However, this increase in acrylamide groups (20 and 30 mg/kg/day) was significantly higher than that in the control mice. As shown in Figure 10, the proinflammatory cytokine levels of IL-1β, TNF-α, IL-6, and IFN-γ were increased whereas the anti-inflammatory cytokine IL-10 was decreased in colitis mice. The changes of these cytokines were significantly intensified in both acrylamide groups as compared with the control mice. The effect of acrylamide on the NF-κB expression and the production of TNF-α, IL-1β, IL-6, and IFN-γ at dose of 30 mg/kg was greater than that at dose of 20 mg/kg. These results propose that chronic acrylamide exposure is able to promote colitis-induced inflammation through the NF-κB pathway and associated production of proinflammatory cytokines in colon tissue.

**Table 1:** Criteria for scoring disease activity index (DAI)

Score	Body weight loss	Stool consistency	Rectal bleeding
0 1 2 3 4	none 1-5 % 6-10 % 11-20 % >20 %	normal loose stool diarrhea	normal slight bleeding gross bleeding

DAI: disease activity index

NO: nitric oxide

NF-κB: nuclear factor kappa B

TNF-α: tumor necrosis factor; IL: interleukin; IFN: interferon

## Discussion

Our study, for the first time, demonstrated that acrylamide exposure could aggravate ulcerative colitis in a mouse model. Smoking and some food components have long been suspected as the main factors contributing to the pathogenesis of colitis. There is some evidence that consumption of certain foods, especially fast food, increases the risk for inflammatory bowel disease (13, 14). Acrylamide has been known as one of the most important food toxicants and is found in high levels in some foods and tobacco smoke. It has been shown that oral intake of acrylamide severely induced intestinal tissue damage in rats (7). It has also been reported that morphology and histology of the intestinal wall were altered by daily intake of acrylamide in mice (9). Another experiment showed that oral administration of acrylamide produced gastric mucosal erosions and inflammatory infiltration in rats (8). 

In our study, colitis induced by acetic acid was used as an experimental model to study the effect of acrylamide on ulcerative colitis. The histopathological features and clinical symptoms of acetic acid-induced colitis are highly similar to those of human ulcerative colitis (11). The clinical signs of ulcerative colitis including weight loss, stool softening, and rectal bleeding were intensified in mice pretreated with acrylamide. The mortality rate was also remarkably increased in colitis mice exposed to acrylamide. Macroscopic evaluation of the colonic tissue revealed that acetic acid-induced colonic mucosal injuries were aggravated in acrylamide-pretreated mice. Histopathological alterations of the colonic tissue including immune cell infiltration, submucosal edema, epithelial damage, and loss of goblet cells were also intensified in colitis mice exposed to acrylamide. In our study aggravating effect of acrylamide on colitis at dose of 30 mg/kg was higher than that at dose of 20 mg/kg. 

Oxidative stress through excessive release of reactive oxygen species is one of the major mechanisms involved in the development of ulcerative colitis. It has been shown that MDA and carbonyl protein content as indicators of oxidative injury were increased in the colon tissue during ulcerative colitis (11, 16-18). GSH, SOD, and catalase are the most important endogenous anti-oxidants against oxidative stress. Many studies have revealed that SOD, GSH, and catalase levels were reduced in colon during experimental ulcerative colitis (10, 11, 19-21). 

It has been proposed that oxidant and anti-oxidant imbalance is associated with the pathogenesis of several diseases and can be triggered by various toxic agents such as acrylamide (22, 23). Acrylamide induces oxidative stress in many tissues by generation of reactive oxygen metabolites. Acrylamide causes intracellular overproduction of free radicals and consequently results in oxidative protein and DNA damage, mitochondrial dysfunction, and inflammation (24). The compound acrylamide and its metabolite glycidamide are detoxified by conjugation with GSH to form the glutathione conjugates which are then excreted in urine (25). Several studies have shown that acrylamide exposure induces lipid and protein oxidation and decreases the free radical scavenging enzymes including SOD, catalase, and GSH in various tissues (7, 22, 26, 27). 

In our study we showed that induction of colitis in mice pretreated with acrylamide further increased the colonic levels of carbonyl proteins and MDA as indicators of oxidative damage. Acetic acid-induced colitis caused a significant reduction in SOD, GSH, and catalase levels in colonic tissue, while colitis induction in acrylamide-pretreated mice intensified the reduction of SOD, GSH, and catalase. These effects of acrylamide were dose dependent. More reduction in SOD, GSH, and catalase levels may also result from consumption of these anti-oxidants by excessively produced superoxide radicals and hydrogen peroxide in colon tissue. These results strongly suggest that exposure to acrylamide generates more free radicals and depletes endogenous anti-oxidants in the colonic tissue. The consequence of this redox imbalance makes colon tissue more susceptible to the initiation and development of ulcerative colitis. 

Overproduction of NO may damage gastrointestinal tissue through inflammatory and oxidative stress processes (28). At high concentrations, NO can act as a free radical and is thought to be involved in the progression of ulcerative colitis. It has been shown that integrity of colon tissue can be affected by the excessive production of NO and peroxynitrite (29, 30). The expression of iNOS can be induced by endotoxins and proinflammatory cytokines (IL-1β and IFN-γ) during ulcerative colitis (31). Several studies have shown that acrylamide significantly induces expression of iNOS and overproduction of NO in different tissues (6-8, 32, 33).

Consistent with previous studies, our findings showed that iNOS expression and NO production in colon were increased in acetic acid-induced colitis. Chronic exposure of mice to acrylamide led to further increase in iNOS activity and higher concentration of NO in colon tissue. 

Intestinal epithelial cells and activated neutrophils can release cytokines and chemokines which have important roles in the migration and infiltration of additional immune cells into inflamed intestinal mucosa (34, 35). MPO and the chemokine MCP-1 are biomarkers of tissue neutrophil infiltration which is used to assess acute inflammation. It has been demonstrated that colonic tissue levels of MPO and MCP-1 were increased in ulcerative colitis (11, 36, 37). Neutrophils and intestinal epithelial cells also produce matrix metalloproteinases (MMPs), which facilitate the recruitment and infiltration of immune cells through degradation of extracellular matrix and release of chemokines and cytokines. MMP-9 is an important member of MMPs, which has been shown to be increased in inflammatory disorders. In ulcerative colitis, excess MMP-9 activity leads to destruction of the intestinal barrier (34, 38, 39). 

In the present study we showed that colitis induction significantly increased MPO, MCP-1, and MMP-9 levels in the colon tissue. Induction of colitis in mice pretreated with acrylamide resulted in an additional elevation in colonic MPO, MCP-1, and MMP-9 levels. In our experiment the effect of acrylamide on MMP-9, MPO, and MCP-1 levels at the dose of 30 mg/kg was greater than that at dose of 20 mg/kg. These findings demonstrate the ability of acrylamide to exacerbate colonic inflammatory response through promoting the activation and infiltration of neutrophils in the colonic mucosa and subsequently disruption of the epithelial barrier integrity. In agreement with this result, histological analysis also revealed that acrylamide-exposure caused more neutrophil infiltration in colitis tissue. Moreover, acrylamide-induced neutrophil infiltration can additionally increase oxidative stress and production of ROS leading to more damage of colonic epithelial cells. Furthermore, acrylamide may influence the permeability of the intestinal epithelium through increasing the production of MMP-9 which is able to degrade extracellular matrix components of the intestinal tissue.

Chronic overexpression of cytokines such as TNF-α promotes apoptosis of intestinal epithelial cells (40). Caspase-3 is an executioner caspase and is considered an important hallmark of apoptosis. DNA damage or oxidative stress can activate caspase-3 in the intestinal epithelium by an intrinsic mitochondrial pathway (41). Caspase-3 has been shown to increase in animal models of ulcerative colitis (42, 43). Mitochondrial stress and dysfunction in the intestinal epithelium play an essential role in the initiation and recurrence of inflammatory bowel disease (44). Excessive ROS can attack the respiratory chain of mitochondria and increase mitochondrial membrane permeability leading to the release of cytochrome-c and activation of apoptotic caspases (45).

In the present study, the levels of cytochrome-c and caspase-3 as markers of apoptosis were significantly and dose-dependently increased in colon tissue of colitis mice pretreated with acrylamide (20 and 30 mg/kg/day). These findings are in agreement with other studies that show an increase in the content of cytochrome-c and caspase-3 activity in acrylamide-induced toxicity (22, 46, 47). Therefore, our results suggest that one of the possible mechanisms by which chronic acrylamide-exposure exacerbates ulcerative colitis could be due to its ability to release mitochondrial cytochrome-c through induction of oxidative stress or DNA damage. Increased cytochrome-c activates caspase-3 protein and induces apoptosis in intestinal cells, which eventually leads to mucosal ulceration. 

The NF-κB signaling cascade is activated in patients with ulcerative colitis and leads to aberrant production 

of inflammatory cytokines and chemokines. (48). It has been reported that activation of NF-κB in epithelial cells and macrophages increases the expression of iNOS and subsequent overproduction of NO in ulcerative colitis (49). NF-κB signaling is involved in the oxidative stress and inflammation induced by acrylamide (5). It has been shown that NF-κB has a key role in acrylamide-induced neurotoxicity (50), hepatotoxicity, and nephrotoxicity (22). 

The present study showed that acetic acid-induced colitis increased NF-κB expression in colon tissue of mice. However, induction of colitis in mice exposed to acrylamide dose-dependently resulted in a higher level of NF-κB expression. Therefore, this finding suggests that exposure to acrylamide can potentiate the activation of NF-κB pathway in colon tissue and lead to aggravation of colonic inflammation. 

Cytokines seem to have a prominent role in the development of clinical symptoms and extra-intestinal inflammatory manifestations of ulcerative colitis, as well as colitis-associated cancer. Moreover, it has been shown that TNF-α and IFN-γ can induce apoptosis of intestinal epithelial cells (11, 51). TNF-α and IL-1β are potent inflammatory mediators, production of which is remarkably increased in the colon tissue of patients with colitis (37). They also mediate the production of MMPs which are involved in the degradation of extracellular matrix and mucosal injury (48). Under inflammatory conditions, IL-6 induces expression of intercellular adhesion molecule (ICAM)-1 which is essential for migration and adhesion of activated leukocytes to the colonic epithelial cells (52). As an important anti-inflammatory cytokine, IL-10 has been revealed to be decreased in numerous mouse models of colitis (53).

It has been reported that acrylamide caused intestinal tissue damage in rats by increasing the expression of IL-1β, TNF-α, and IL-2 (7). It has also been reported that TNF-α, IL-6, and IL-1β levels were increased in acrylamide-induced hepatotoxicity and nephrotoxicity (22, 54). 

In accordance with other studies, we showed that colitis induction resulted in a significant increase in the level of proinflammatory cytokines (IL-1β, TNF-α, IL-6, and IFN-γ) and decreased the anti-inflammatory cytokine IL-10 in colon tissue. Our data showed that these cytokines in colon tissue were increased in colitis mice exposed to acrylamide. The effect of acrylamide on IL-1β, TNF-α, IL-6, and IFN-γ levels at the dose of 30 mg/kg was greater than that at the dose of 20 mg/kg. Acrylamide-induced toxicity intensified the imbalance between pro and anti-inflammatory cytokines released in colon. It seems that the production of inflammatory mediators is an essential mechanism of acrylamide for aggravation of colon inflammation during development of ulcerative colitis. It can be suggested that exposure of intestinal epithelial cells to acrylamide leads to overproduction of various proinflammatory cytokines, which have been shown to damage tight junction of intestinal epithelial cells. This effect of acrylamide may lead to epithelial barrier dysfunction and ultimately aggravate mucosal inflammation and ulceration in colitis. 

## Conclusion

In summary, this study revealed that oxidative stress and inflammation induced by acrylamide can accelerate the induction of ulcerative colitis and aggravate colonic mucosal injury during the progression of the disease. We found that acrylamide is able to impair the anti-oxidant defense mechanism, induce apoptosis and increase inflammatory cytokines and NF-κB activity which play a fundamental role in the pathogenesis of colitis. In conclusion we propose that, chronic acrylamide exposure from food (or smoking) may increase the risk of ulcerative colitis. Further research is needed to investigate the effect of acrylamide in patients with inflammatory bowel diseases.
